# Structural interactions of TLP18.3 and Psb27-H1 to the luminal CP43 and rubredoxin-ENH1 to the stromal side of photosystem II in higher plants

**DOI:** 10.1016/j.jbc.2026.111363

**Published:** 2026-03-10

**Authors:** Haijun Liu, Youngwoo Lee

**Affiliations:** Department of Biology, Saint Louis University, St Louis, Missouri, USA

**Keywords:** cross-linking mass spectrometry (XL-MS), photoprotection, photosystem II assembly, Psb27, Psb32, PSII assembly, redox regulation, rubredoxins, TLP18.3

## Abstract

Thylakoid lumen protein 18.3 and Psb27 are known proteins on the luminal side of photosystem II (PSII). The structural locations of these two proteins are still absent in the currently available higher plant PSII cryogenic electron microscopy structures. We interrogated the structural locations of these proteins using chemical cross-linking followed by LC-MS/MS analysis. Structural mass spectrometry results then provided chemical restrains to direct structural modeling to determine the collective binding/stabilization of these two proteins to the luminal PSII CP43 protein. Using this pipeline, we also found the structural location of a rubredoxin protein on the stromal side of PSII. Discovery of this redox active iron-sulfur protein in the vicinity of PSII subunit D1/D2 proteins greatly showcases the importance of the redox processes that are potentially involved in PSII assembly or less known steady-state functionality or photoprotection. This structural mass spectrometry platform highlights its powerful applicability in protein complex discovery.

Photosystem II (PSII) is one of the two major photosystems in oxygenic photosynthesis. It is the first protein complex in the light reactions of plant, algae, and cyanobacteria, and plays a central role in splitting water and initiating the electron transport (1, 2, 3, 4). PSII is highly efficient, but it is also the most vulnerable part of the photosynthetic machinery due to its demanding photochemistry. Because PSII is constantly damaged by light, plants, algae, and cyanobacteria have evolved multiple photoprotection mechanisms (5, 6, 7) and a relatively conserved and dedicated repair cycle (8, 9, 10). This keeps photosynthesis running efficiently under variable light and other environmental changes.

PSII is a pigment–protein complex comprising more than 20 subunits (4, 11, 12, 13), along with many pigments and other cofactors. In addition, PSII binds peripheral antenna proteins that increase the light harvesting cross-section as well as dissipate excess energy (14, 15, 16, 17). PSII repair, biogenesis, and assembly in plants and cyanobacteria is a highly dynamic, multistep process that involves a whole network of factors beyond just the core subunits (8, 18). PSII regeneration works as an assembly line, with proteases chopping out damaged D1, insertases/chaperones placing the new one, and a set of auxiliary proteins (HCFs, LPAs, Psb27/Psb28/Psb29, RubA, PAM68, and OHPs) ensuring that every step goes back together efficiently. Current structural knowledge on PSII assembly and repair factors is much more advanced in cyanobacteria than in higher plants by taking advantages of the genetically tractable systems, easily purified PSII assembly intermediates, and large amounts of material from bacteria cell culture. The recent development of cryogenic electron microscopy (cryo-EM) techniques plays an essential role in elucidating the assembly intermediates (19, 20, 21, 22, 23, 24). It should be noted that while most progress has been made in cyanobacteria, the structural studies of their counterparts in higher plants remain to be further explored. For example, thylakoid lumen protein 18.3 (TLP18.3, AT1g54780 in *Arabidopsis thaliana* and *sll1390* in *Synechocystis* sp. PCC 6803 among others) has been known involved in PSII repair cycle (25, 26, 27), particularly in dephosphorylation (28) or turnover of damaged D1 protein. However, the structural location of this protein remains unclear.

Rubredoxin (Rub) is a redox active protein supposedly located on the stromal side (RBD1, AT1g54500 in *A. thaliana* and RubA, *slr2033* in *Synechocystis* sp. PCC 6803). It is essentially required for early PSII assembly and stability (29, 30, 31, 32). It specifically ensures the insertion/stabilization of the non-heme Fe in the PSII reaction center (D1/D2). However, their structural locations in PSII remain unclear.

Cross-linking mass spectrometry (XL-MS) is a structural proteomics technique used to study protein–protein interactions and protein conformations at residue-level resolution (33, 34). It has become a powerful complement to cryo-EM, X-ray crystallography, and NMR, especially for dynamic or heterogeneous, low-abundant protein complexes. In a cross-linking reaction, a bifunctional chemical cross-linker reacts with two amino acid side chains (commonly Lys–Lys, Lys–Ser/Thr/Tyr if NHS-ester–based cross-linker is used, Asp/Glu, or even cysteines). This forms a covalent bridge that captures proteins in their native or near-native conformation. Usually, there are several types of links resulting from the reaction: interprotein (between different proteins), which is useful for mapping protein interfaces, and intraprotein (within one protein), which gives distance restraints for folding and is useful for the justification of *in silico* 3D protein structures. After protease digestion, small population of cross-linked peptide species (usually with two N-termini and two C-termini) is formed among the uncross-linked regular single peptide which contain one N terminus and one C terminus. Liquid chromatography-tandem mass spectrometry (LC-MS/MS) is used to detect the cross-linked peptides. Specialized MS2/MS3 fragmentation helps identify the linked sites. Cross-linkers with MS-cleavable bonds (*e.g.,* DSSO, DSBU) produce distinctive fragment patterns that simplify identification. Data analysis is a process of using dedicated search engines, *e.g.,* pLink (35), Kojak (36), StavroX (37), MeroX (38), and XiSearch (39) to interpret the spectra.

Protein cross-linking and its combinational use with LC-MS/MS, commonly termed XL-MS, in recent years has been very successful (40, 41, 42). It captures weakly or transiently bound subunits during protein complex life cycle in the aqueous phase, greatly expanding the scope of detecting protein complex structure (43, 44). In this research, we have used isotopically encoded cross-linker and XL-MS to capture the structural location of several PSII assembly/stability factors within higher plant PSII preparations. Remarkably, we identified that Plant Psb27-H1 is cross-linked to CP43, and TLP18.3 is cross-linked to Psb27 on the luminal side. Interestingly, we identified a Rub-like protein encoded by ENH1 (A0A9R0ICQ3, hereafter so-Rub-ENH1) is cross-linked to PsbE on the stromal side. In *Arabidopsis*, Rub-ENH1 (AT5G17170) functions in ion homeostasis and detoxification of the reactive species (45, 46). Please note that, both AT5G17170 (ENH1) and AT1G54500 (RBD1) contain a “Rub” domain, they are functionally distinct with the latter attracted lots of research (5, 29, 30, 31, 47, 48). Using our MS)-identified cross-links as distance restraints and protein–protein docking software, we were able to generate Psb27–TLP18–CP43 PSII structural model. Using similar strategy, we proposed the structural model of Rub-ENH1–PSII. By combining with the absolute quantitative MS results of those involved proteins from cyanobacteria, we propose diverse functional and nonfunctional PSII populations with varied occupancy of their binding partners in steady state and assembly states in PSII life cycle.

## Results and discussion

In our XL-MS searching pipeline, spinach PSII samples without chemical cross-linking treatments were first subjected to LC-MS/MS analysis and detection against the National Center for Biological Information whole spinach proteome (*Spinacia oleracea*, taxonomy id: 3562, 67,364 proteins). The resulting identified protein list (∼200 proteins, .fasta file) was then used as the searching database for the LC-MS/MS result (Thermo Fisher Scientific, .raw files) of PSII samples treated with cross-linking reactions, *i.e.*, BS3-H_12_/D_12_ (1:1). Searching was performed on pLink three software (35). Notably, many cross-links (inter/intraproteins), loop-links, and mono-links were identified, such as cross-links between CP43-PsbO which can serve as a control since the close association between them has been elucidated in fully assembled higher plants PSII (15, 49, 50, 51, 52). Here, we focus on the PSII auxiliary protein components, the structural locations of which have remained unclear (8).

**Figure 1:**
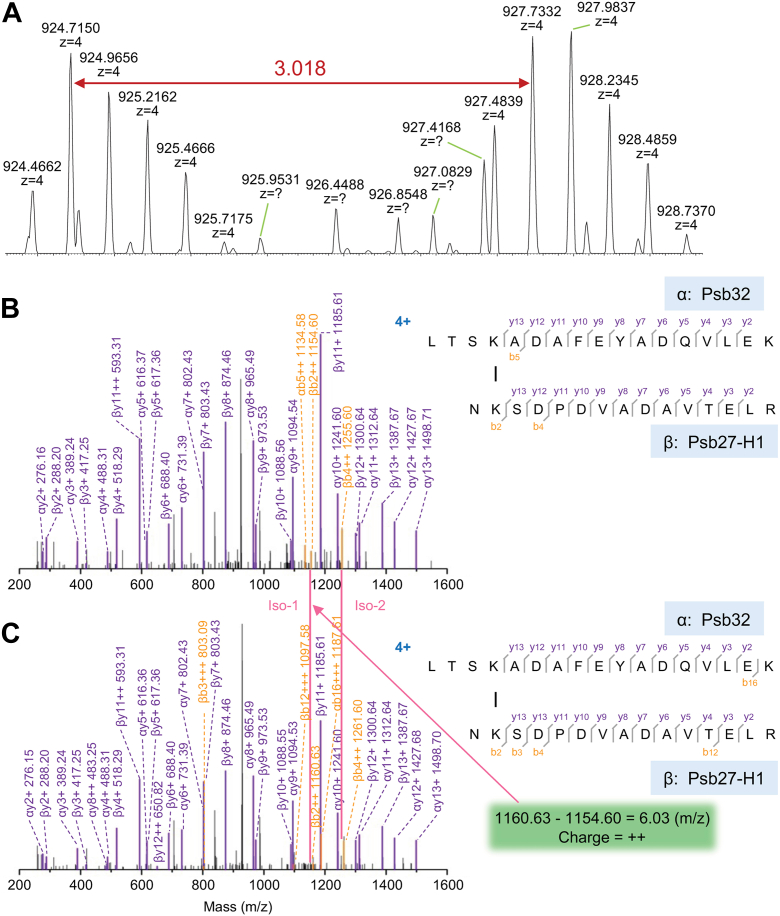
**MS and MS/MS spectra of cross-link between Psb32 and Psb27-H1.***A*, MS spectra of precursor ion for the light and heavy cross-linked species (BS3-H_12_/D_12_), displaying two envelopes with similar intensity, separated by *m/z* 3.018 (z = 4). *B*, product-ion spectrum of the cross-linked peptides with BS3-H_12_. *C*, product-ion spectrum of the cross-linked peptides with BS3D_12_. α, *top* peptide of Psb32; β, *bottom* peptide of Psb27-H1. The product-ion pairs with isotopic fingerprint are exemplified by an ion pair βb2++ with *m/z* difference of 6.03 (*green box*) referenced by many almost identical ion pairs in (*B*) and (*C*), *e.g.,* αy9 = 1094.54 (*B*) *versus* 1094.53 (*C*). MS, mass spectrometry; MS/MS, tandem mass spectrometry.

Table 1 lists all the relevant cross-linking pairs to be reported and discussed in this research. First, we identified that K^144^ of TLP18.3 (gene ID A0A9R0JN50, uniprot.org) is cross-linked to K^92^ of Psb27-H1 (A0A9R0KAU1) (Fig. 1). The precursor-ion (charge 4+) of this cross-link appears as a doublet envelope (1:1) owing to the isotopic coding of the cross-linker BS3-H_12_/D_12_ (premixed with 1:1 ratio) used in the experiment (Fig. 1*A*). The corresponding doublet peaks (envelope) show an *m/z* 3.018 mass shift. The product-ion coverage is 75% and 25% for y ions and b ions, respectively (Fig. 1, *B* and *C*). Remarkably well-identified series y ions in peptide LTSK^144^ADAFEYADQVLEK from TLP18.3 and NK^92^SDPDVADAVTELR from Psb27-H1 clearly confirm their identities. Even in MS2 spectra, both precursor ions (light 924.48, 4+; heavy 927.50, 4+) that were not fragmented were also identified (Fig. 1, *B* and *C*). The exemplified product-ion pairs (charge 2+) with isotopic features are ßb2 and ßb4 with *m/z* shift of 6.03 and 6.04, respectively, referenced by many identical y ions independent of the use of BS3-H_12_/D_12_ cross-linker (Fig. 1*C*), unequivocally supporting the cross-linking site occurring in the assigned location, *i.e.*, TLP18.3-K^144^ and Psb27-H1-K^92^. The criteria used in this example will be applied throughout this article to justify if a cross-link is a confident one or not. Identification of the cross-link TLP18.3-K^144^–Psb27-H1-K^192^ indicates that the two primary amines from these two Ks must be solvent-accessible and the spatial distance between them has to be in the range of <30 Å ensuring cross-linking chemistry reactions to occur since the cross-linker arm span of BS_3_ is ∼11.4 Å (molecular vibration, stretching, *etc.*, have to be considered). Due to these features, the identified cross-linking species between two peptide fragments can be considered as an experimental restraint to justify *in silico* models or models generated using other biophysical tools, such as X-ray crystallography or cryo-EM (40, 53, 54).

**Table 1:** Identification of protein cross-linking in photosystem II (spinach)

Proteins (Site-to-Site)	Protein (UniProt-ID)	Precursor mass (*m/z*)	Peptide	Linker	*z*	E value	Score
CP43 (382)-PsbO (133)	P06003-P12359	2359.207531	KDLQPWQER(1)-KFCLEPTK(1)	BS3	4	0.989499	−0.007282
		2371.285607	KDLQPWQER(1)-KFCLEPTK(1)	BS3D_12_	4	0.986468	−0.00384
TLP18.3 (144)-Psb27-H1 (92)/	A0A9R0JN50-A0A9R0KAU1	3694.847063	LTSKADAFEYADQVLEK(4)-NKSDPDVADAVTELR(2)	BS3	4	0.998777	0.013826
		3706.918791	LTSKADAFEYADQVLEK(4)-NKSDPDVADAVTELR(2)	BS3D_12_	4	0.998734	0.004036
Psb27-H1 (167)-CP43 (382)/	A0A9R0KAU1_-P06003	2982.558519	LLEEMDSVEKALLR(10)-KDLQPWQER(1)	BS3	4	0.898488	−0.001825
		2994.619415	LLEEMDSVEKALLR(10)-KDLQPWQER(1)	BS3D_12_	4	0.776851	−0.022447
Psb27-H1 (167)-Psb27-H1 (78)/	A0A9R0KAU1-A0A9R0KAU1	2716.464003	LLEEMDSVEKALLR(10)-ETKEVLSK(3)	BS3	4	0.981825	−0.004742
		2728.538023	LLEEMDSVEKALLR(10)-ETKEVLSK(3)	BS3D_12_	4	0.979781	−0.01224
Psb27-H1 (92)-Psb27-H1 (83)/	A0A9R0KAU1-A0A9R0KAU1	2597.377531	NKSDPDVADAVTELR(2)-EVLSKVR(5)	BS3	4	0.899955	−0.000431
		2609.449167	NKSDPDVADAVTELR(2)-EVLSKVR(5)	BS3D_12_	4	0.822458	−0.010313
Rubredoxin (185)-PsbE (2)/	A0A9R0ICQ3-P69383	1633.836901	KLSEAQK(1)-SGSTGER(1)	BS3	3	0.99242	−0.002177
		1645.913341	KLSEAQK(1)-SGSTGER(1)	BS3D_12_	3	0.74715	−0.007255

Listed are protein names, protein ID from uniprot.org, precursor mass of the cross-linked species, peptide sequence, link used (isotopically encoded, BS3 *versus* BS3D_12_), charge state of the precursor ion, and evaluation of the scoring of the identification. The cross-linked residues are labeled with their respective residue numbers in parentheses.

**Figure 2:**
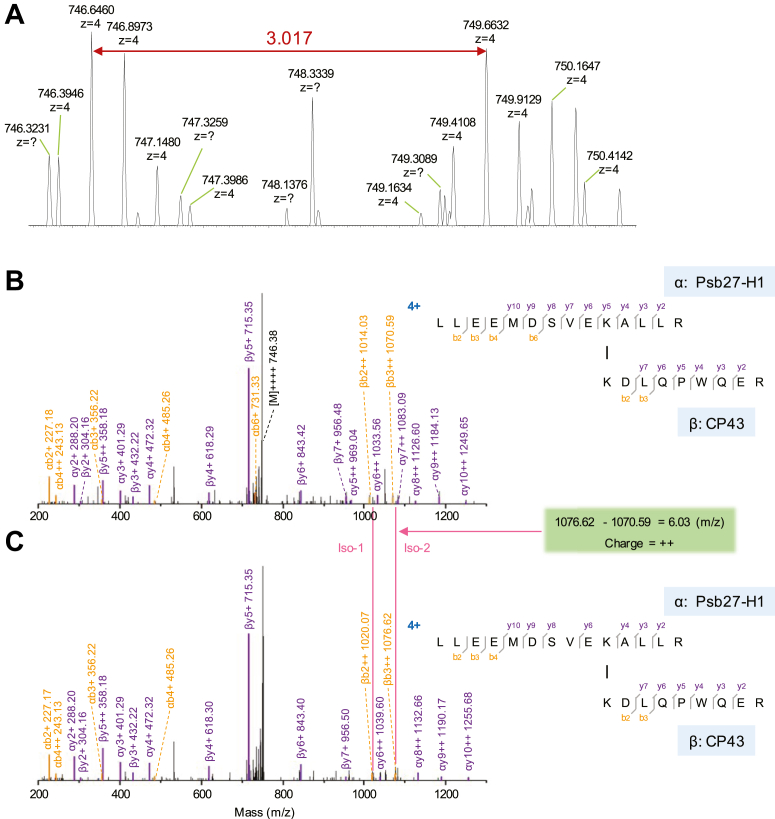
**MS and MS/MS spectra of cross-link between CP43 and Psb27-H1.***A*, MS spectra of precursor ion for the light and heavy cross-linked species (BS3-H_12_/D_12_), displaying two envelopes with similar intensity, separated by *m/z* 3.017 (z = 4). *B*, product-ion spectrum of the cross-linked peptides with BS3-H_12_. *C*, product-ion spectrum of the cross-linked peptides with BS3D_12_. α, *top* peptide of Psb27-H1; β, *bottom* peptide of CP43. The product-ion pairs with isotopic fingerprint are exemplified by an ion pair βb3++ with *m/z* difference of 6.03 (*green box*) referenced by many almost identical ion pairs in (*B*) and (*C*), *e.g.,* βy5 + = 715.35 in both (*B*) and (*C*). MS, mass spectrometry; MS/MS, tandem mass spectrometry.

Figure 2 shows the cross-link between Psb27-H1 and CP43, one of the core antennas of PSII. Psb27-H1-K^167^ in peptide LLEEMDSVEK^167^ALLR is cross-linked to CP43-K^382^ of peptide K^382^DLQPWQER. Psb27-H1-K^167^ and CP43-K^382^ are located in Psb27 helix 4 and CP43 loop E, respectively. Briefly, all MS1 and MS2 spectra, particularly their isotopic features, meet our above-mentioned criteria of a confident cross-link, *i.e.*, high percentage peptide sequence coverage, isotopic doublet of MS1, and some fragmented ion pairs (or production ion pairs) which contain the isotopically encoded BS3-H_12_/D_12_. Psb27 has been shown to copurify with PSII complexes in a number of early biochemical preparations in different cyanobacteria (12, 55, 56). Close association of Psb27 to CP43 has been proposed in the literature in cyanobacteria using XL-MS (41, 57) and biochemical analysis (58). MS-assisted protein footprinting also confirmed and elucidated the dynamic interactions of Psb27 with CP43, specifically, loop E of CP43, in the presence and absence of a fully process D1 protein using a series of isolated PSII mutant complexes (59, 60). Recent cryo-EM analysis showed more structural details that Psb27 is associated with CP43 at the luminal side through helix 2 and helix 3 of Psb27 and a loop region between helix 3 and helix 4 of CP43 (loop C) as well as the large, lumen-exposed, and hydrophilic E-loop of CP43 in *Thermosynechococcus vulcanus* (20). A PSII assembly intermediate containing Psb27, Psb28, Psb43 further demonstrated some key features of both PSII acceptor and donor (Mn4CaO5) sides using cryo-EM single particle analysis (19), remarkably advancing the understanding of PSII assembly mechanism.

Psb27 in *Arabidopsis* (AtPsb27) has been shown to function in PSII repair and light acclimation (61, 62). A crystal structure of *Arabidopsis* Psb27 also exists (63), but it has not been copurified/cocrystallized with PSII preparations or mapped onto PSII assembly intermediates. Its exact binding site or interaction with CP43 has not been experimentally resolved. We consider our results using spinach PSII particles (Fig. 2) are the first evidence supporting the close association of Psb27 and CP43 in higher plants. In spite of the low sequence similarity (57%) of the cyanobacterial Psb27 and the higher plant counterpart Psb27, both proteins apparently form a 4-helix bundle structure (63, 64, 65, 66), possibly indicative of its conserved binding site.

Using HADDOCK (67), we were able to dock spinach Psb27-H1 generated by AlphaFold 3 (68) onto spinach CP43 luminal side–CP43 structure which was extracted from a recent spinach PSII structure (PDB ID: 8Z9D) (15). Briefly, HADDOCK clustered 167 structures in five cluster(s), which represent 83% of the water-refined models HADDOCK generated. The statistics of the top five are shown in Table 2. The top cluster is the most reliable models according to HADDOCK scoring system. Its Z-score measures how well a particular cluster of docking models perform relative to all other clusters generated in the same run, usually the more negative, the better. Basically, the software takes several parameters into consideration during the *in silico* computation, such as cluster size, RMSD from the overall lowest energy structure, Van der Waals energy, electrostatic energy, desolvation energy, restraints violation energy, and buried surface area. Cluster 3 seemingly excels from multiple parameters screening, as indicated by both electrostatic energy (−355.7 kcal/mol) and Van der Waals energy (−36.5 kcal/mol). The buried surface area was calculated as 1638.4 Å^2^ (Table 2).

**Table 2:** HADDOCK docking results of CP43 and Psb27-H1

Cluster	3	1	4	5	2
HADDOCK score	−104.4 ± 2.1	−92.0 ± 1.9	−78.9 ± 4.5	−61.9 ± 7.9	−61.0 ± 1.3
Cluster size	25	89	8	7	38
RMSD^a^	3.4 ± 0.5	1.4 ± 0.3	2.3 ± 0.5	3.7 ± 0.4	2.6 ± 0.8
Van der Waals energy	−36.5 ± 3.8	−33.9 ± 3.2	−32.2 ± 2.2	−17.6 ± 4.7	−13.5 ± 2.5
Electrostatic energy	−355.7 ± 9.4	−320.2 ± 35.6	−296.5 ± 26.5	−240.3 ± 22.0	−279.3 ± 16.0
Desolvation energy	3.2 ± 3.9	5.7 ± 3.5	11.9 ± 4.5	2.3 ± 1.6	7.4 ± 0.8
Restraints violation energy	0.8 ± 0.8	1.7 ± 1.6	6.5 ± 9.0	14.5 ± 14.6	9.7 ± 11.1
Buried surface area	1638.4 ± 129.8	1557.0 ± 108.7	1513.7 ± 61.4	1053.1 ± 167.0	999.8 ± 85.4
Z-score	−1.5	−0.7	0	1	1.1
Distances (Å)^c^	27	23.275	19.075	31.5	26.775
RMSD with cyano^b^	1.03	1.06	1.05	0.99	1.08

a RMSD from the overall lowest-energy structure.

b RMSD from the cyanobacterial CP43-Psb27 (PDB ID: 7NHP).

c Average distances (Å) (α-carbon).

**Figure 3:**
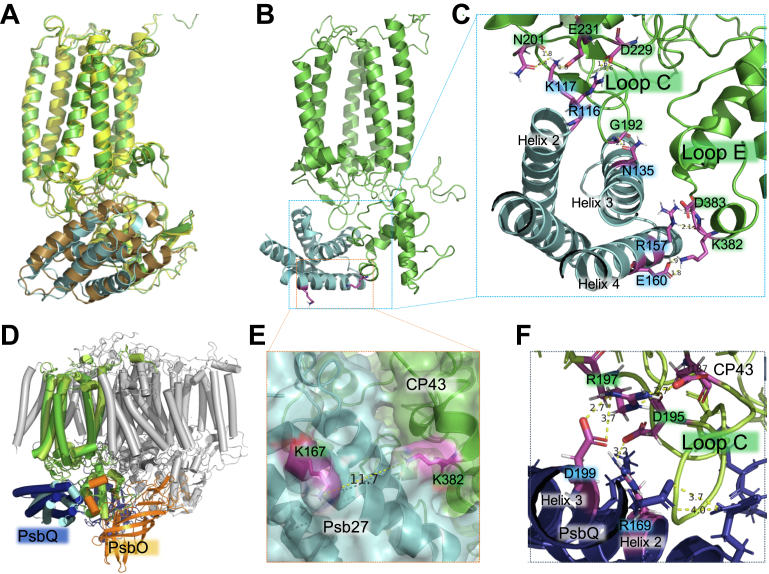
**Structural model of luminal Psb27-H1 and CP43 interaction.***A*, alignment of cyanobacterial CP43–Psb27 with higher plant CP43–Psb27-H1 model. Cyanobacterial CP43 (*yellow*) and Psb27 (*olive*). *B*, close association of CP43 luminal loops with Psb27-H1. *C*, amino acid residues involved in salt-bridges between CP43 and Psb27-H1. *D*, spatial conflicts of PsbQ and Psb27-H1. *E*, solvent accessibility of amino acids involved in chemical cross-linking and the distance. *F*, salt-bridges between CP43 and PsbQ *versus* CP43 and Psb27-H1 in (*C*). Color code: CP43 (*green*), Psb27-H1 (*cyan*), TLP18.3 (*wheat*), and PsbQ (*dark blue*). TLP, thylakoid lumen protein.

The structural location of Psb27-H1 in our CP43-Psb27 model shows high similarity to that of the cyanobacterial CP43-Psb27 models (19, 20, 69) with RMSD of ∼ 1 Å (Fig. 3*A*). BS3, used in this research, is a homobifunctional NHS-ester cross-linker which is targeted to primary amines (-NH2), mainly the ε-amino group of lysine side chains and the α-amino group at protein N termini. Basically, the amine groups need to be solvent accessible for BS3 to reach them, buried lysine side chains prevent cross-linking reaction occurring. In practical terms, only solvent accessible lysine side chains or N termini within ∼30 Å Cα–Cα distance without apparent spatial conflicts (when considering side-chain flexibility + linker length) will cross-link. Solvent accessibility and spatial conflicts/distances comprise the key criteria to be used in XL-MS guided protein structure justification. In our model, the distance of two cross-linked lysine residues (Fig. 2) is measured as 11.7 Å in cluster 3 model 4, and they are solvent accessible (Fig. 3, *B* and E), highly consistent with the cross-linking chemistry of BS3 (11.4 Å arm span) if molecular vibration and stretching are considered. Please note the α-carbon distance of the model is 17.8 Å (not shown). There are four salt-bridges between CP43 and Psb27-H1 in this model. Two salt-bridging pairs seemingly stabilize the interaction between CP43 (loop C) and Psb27-H1 (helix 2): Psb27-H1-K^117^–CP43-E^231^ and Psb27-H1-R^116^–CP43-D^229^. Other two salt-bridging bonds are located on Psb27-H1 helix 4 and CP43 loop E: Psb27-H1-R^157^–CP43-D^383^ and Psb27-H1-E^160^–CP43-K^382^ (Fig. 3*C*). Hydrogen-bonds are also observed in the interface between these two proteins (Fig. 3*C*). Cyanobacterial Psb27 is lipid-modified, and this lipid anchoring enhances the stability of Psb27–CP43 interaction (56, 70). Lipid modification/stabilization of higher plant Psb27 has been observed. The structural location of Psb27-H1 is apparently in conflict with that of PsbQ in higher plant PSII, but not in conflict with that of PsbO (Fig. 3*D*), which is slightly different than the situation of cyanobacterial PSII. Instead of having four salt-bridges between CP43 and Psb7-H1, there are only two between CP43 and PsbQ: PsbQ-D^199^–CP43-R^197^ and PsbQ-R^169^–CP43-K^195^, possibly indicative of less binding affinity of PsbQ to PSII in this region. However, PsbQ has a long unstructured N-terminal extension which is involved in binding/stabilization to PsbP among other PSII subunits (5), providing further stabilization of PsbQ to other PSII components, which may replace the function of the lipid anchor in cyanobacterial Psb27-containing PSII. Both residues of CP43 involved in salt-bridges with PsbQ are located on loop C. Protrusion of CP43 loop C against Psb27 helix 2 and helix 3 is conserved in cyanobacterial PSII and higher plant PSII (Fig. 3, *B* and C). CP43 loop C is also seemingly involved in the interaction with PsbQ through helix 2 and helix 3 in higher plant PSII, the orientation of the helices, however, is different from that of Psb27-H1 (Fig. 3, *C* and *F*).

**Figure 4:**
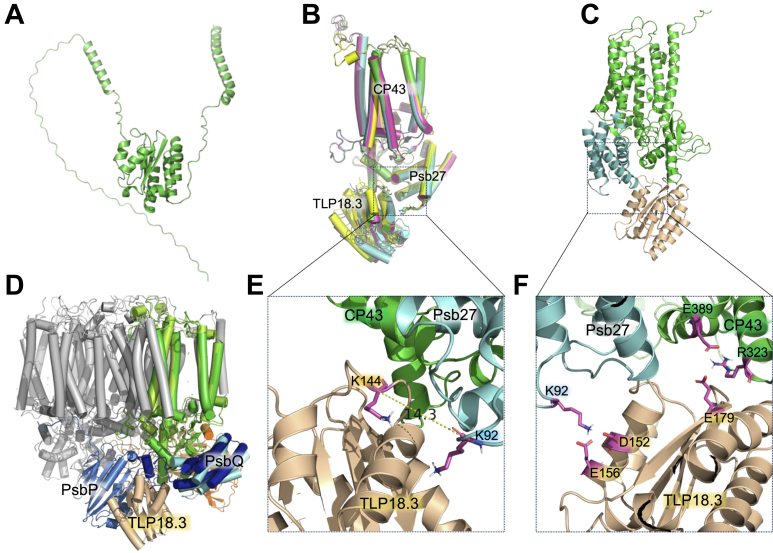
**Structural model of luminal TLP18.3 and CP43–Psb27-H1 interaction.***A,* structural model of TLP18.3 (AlphaFold 3). *B*, four models in cluster 1, showing similar binding location of TLP18.3 TPM domain against Psb27-H1 and CP43. *C*, model 1 in cluster 1. *D*, structural conflicts of TLP18.3 with PsbP. *E*, α-carbon distance between the cross-linked amines in Psb27-H1 and TLP18.3, respectively. *F*, cregions of TLP18.3 and Psb27, highlighting salt-bridges. Color code: CP43 (*green*), Psb27-H1 (*cyan*), TLP18.3 (*wheat*), PsbQ (*dark blue*), PsbP (*marine*), and other PSII subunits (*gray*). TLP, thylakoid lumen protein.

The same structural prediction and justification practice were then applied to TLP18.3 and CP43-Psb27-H1 complex, which has been justified (Fig. 3*B*). HADDOCK clustered 106 structures in 14 cluster(s), which represent 53% of the water-refined models HADDOCK generated. TLP18.3 is considered as the founding protein of TPM domain family which is called the thylakoid acid phosphatase domain and has a Rossmann-like fold characterized by alpha-helical fold and flanking four central β strands (Fig. 4*A*). The TPM fold has not been found in other protein domains to date (25, 27, 28). The top cluster 1 generated by HADDOCK server containing four models is the most reliable one according to the HADDOCK scoring system (Table 3 and Fig. 4, *B* and C). According to this model (cluster 1 model 1), TLP18.3 interacts with Psb27-H1 through two regions: TLP18.3-E^156^/D^152^ is in salt-bridging with Psb27-H1-K^92^ and CP43-R^323^/E^389^ is in close contact with TLP18.3-E^179^ (Fig. 4, *C* and F). TLP18.3, however, is in spatial conflict with PsbP on the luminal side (Fig. 4*D*). During our manuscript preparation, a preprint report indicated that the close association of Psb32 (homolog of TLP18.3 from cyanobacteria) with Psb27 in cyanobacteria (71). We indeed took advantage of this preprint publication and incorporated the contact regions as the reference binding site to assist the modeling on HADDOCK server. We did not include the structural distance restraints from our MS observation (Fig. 1), aiming to justify the models after the structural modeling is finished. In our following evaluation process, it seems that the distance (14.3 Å α–α carbon *versus* 8.3 Å amine–amine) of the cross-linked species is well in the range of the cross-linking chemistry (Fig. 4*E*). Interestingly, another preprint article also reported the same cross-link site of TLP18.3 and Psb27-H1 using *Arabidopsis* plant (72) as that of our spinach data. The robust cross-linking platform they have built relies on the affinity enrichment technology. Altogether, it seems that the cryo-EM structural studies in cyanobacteria (71), MS studies in another group using *Arabidopsis* (72), and our MS research using spinach all independently demonstrate the structural information of TLP18.3 and Psb27 in PSII.

**Table 3:** HADDOCK docking results of TLP18.3 and CP43–Psb27-H1 complex

Cluster	1	8	12	3	5	14	10	13	2	11
HADDOCK score	−75.1 ± 2.5	−68.0 ± 7.1	−67.3 ± 6.9	−66.3 ± 9.4	−66.0 ± 13.2	−62.4 ± 7.0	−56.9 ± 9.5	−52.5 ± 16.1	−49.0 ± 1.2	−47.2 ± 3.0
Cluster size	29	6	5	8	7	4	5	4	9	5
RMSD^a^	9.3 ± 0.5	10.1 ± 0.4	11.0 ± 0.2	9.5 ± 0.2	7.6 ± 0.8	10.5 ± 0.2	8.9 ± 0.2	1.9 ± 0.6	5.1 ± 0.7	7.0 ± 0.3
Van der Waals energy	−20.5 ± 4.3	−27.9 ± 2.6	−28.8 ± 6.2	−21.3 ± 6.7	−19.0 ± 9.9	−23.4 ± 4.6	−30.7 ± 9.3	−11.2 ± 14.0	−15.2 ± 4.5	−18.0 ± 2.2
Electrostatic energy	−298.8 ± 14.1	−207.8 ± 41.8	−210.9 ± 49.6	−236.4 ± 28.4	−306.3 ± 63.2	−211.4 ± 64.3	−142.1 ± 23.1	−216.5 ± 57.1	−200.5 ± 44.4	−131.1 ± 24.4
Desolvation energy	4.8 ± 3.1	0.6 ± 5.0	2.6 ± 1.7	2.0 ± 3.2	11.5 ± 1.4	2.3 ± 2.4	−1.1 ± 2.9	0.7 ± 4.2	3.6 ± 5.1	−3.8 ± 4.1
Restraints violation energy	3.8 ± 3.4	8.4 ± 10.8	11.1 ± 10.7	4.2 ± 0.3	28.0 ± 15.5	9.5 ± 12.4	33.1 ± 2.4	12.2 ± 20.3	26.7 ± 24.8	8.5 ± 9.6
Buried surface area	1120.2 ± 71.1	1197.4 ± 186.5	1132.7 ± 70.5	1169.4 ± 153.0	1176.1 ± 186.7	1016.9 ± 85.7	1247.5 ± 127.3	986.1 ± 240.6	857.3 ± 32.5	983.2 ± 60.4
Z-score	−1.6	−0.8	−0.7	−0.6	−0.6	−0.2	0.5	1	1.4	1.6

a RMSD from the overall lowest-energy structure.

**Figure 5:**
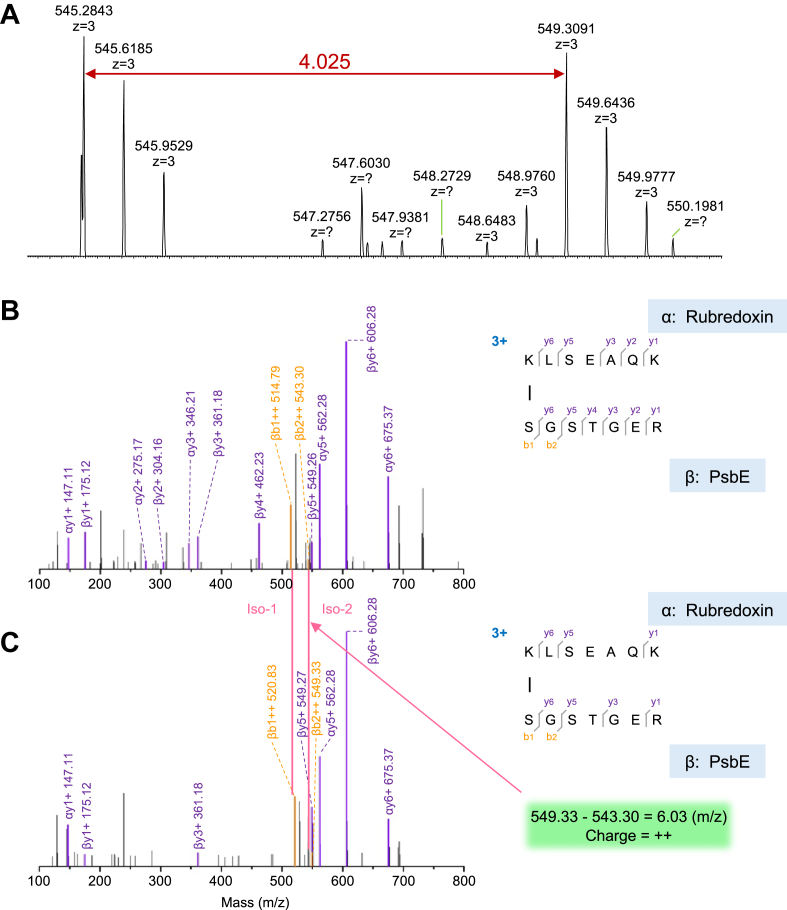
**MS and MS/MS spectra of cross-link between rubredoxin and PsbE.***A*, MS spectra of precursor ion for the light and heavy cross-linked species (BS3-H_12_/D_12_), displaying two envelopes with similar intensity, separated by *m/z* 3.018 (z = 4). *B*, product-ion spectrum of the cross-linked peptides with BS3-H_12_. *C*, product-ion spectrum of the cross-linked peptides with BS3-D_12_. α, top peptide of rubredoxin; β, bottom peptide of PsbE. The product-ion pairs with isotopic fingerprint are exemplified by an ion pair βb2++ with *m/z* difference of 6.03 (*green box*) referenced by many identical ions in (B) and (C), *e.g.,* αy6 + = 675.37. MS, mass spectrometry; MS/MS, tandem mass spectrometry.

Figure 5 shows the cross-link between Rub-ENH1 (A0A9R0ICQ3) and PsbE (P69383), one of the PSII core subunits. Rub-ENH1-K^185^ in peptide LSEAQK^185^AR is cross-linked to the N-terminal primary amine of PsbE S^2^GSTGER (first Met removed after posttranslational modification). Briefly, the precursor (MS1) of the cross-linked species is 3+ charged *versus* more commonly observed cross-linked peptides with two N-terminal primary amines intact which is usually 4+ (Figs. 1 and 2). Both MS1 and MS2 features of the Rub and PsbE cross-link indicate that it is a confident cross-link: high percentage peptide sequence coverage, isotopic doublet of MS1 as well as some fragmented ion pairs (or production ion pairs) due to the use of the isotopically encoded BS3-H_12_/D_12_.

**Figure 6:**
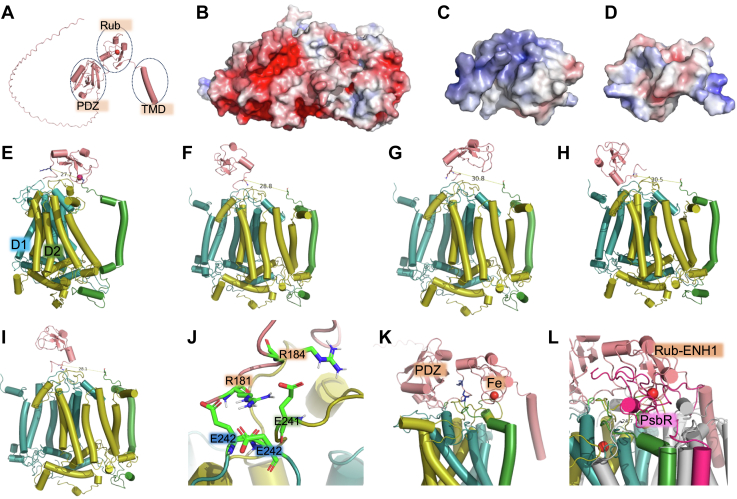
**Structural model of stromal side association of Rub-ENH1 with PSII.***A,* structural model of Rub-ENH1 (AlphaFold 3) with three domains. *B*, electrostatic potential of D1-D2 stromal side. *C* and *D*, Rub-ENH1 rubredoxin non-heme iron domain and back domain, respectively. *E–I,* representative models from top 5 clusters in HADDOCK structural prediction. *J*, close contact between Rub-ENH1 Rub domain and the stromal side of D1-D1. *K*, Rub-ENH1 with three domains sitting on D1-D2. *L*, spatial conflicts between Rub-ENH1 (*salmon*), PsbR (*hot pink*), D1 (*deep teal*), and D2 (*olive*).

Rub was first described in the 1960s (73). In cyanobacteria, green algae, and higher plants, Rub (RBD1, RBD1-like) proteins are thylakoid-localized and play key roles in PSII assembly and repair (29, 30, 31, 32, 47, 48). Loss-of-function mutants show severely impaired PSII activity and blocked PSII assembly as early as in the translation of D1 protein (31, 48). Both cofractionation of RBD1 with PSII RC intermediates and co-immunoprecipitation are direct biochemical evidence supporting that RBD1 closely interacts with PSII components, specifically D1 (32); the structural evidence, however, remains unclear. Here, we report a rubredoxin family protein ENH1 or Rub-ENH1 (45) (spinach, UniProt ID: A0A9R0ICQ3) is closely associated with PSII on the stromal side. Rub-ENH1 is a chloroplast-localized protein with a PDZ domain at the N-terminal region and an Rub domain in the C-terminal part (Fig. 6*A*). Briefly, a PDB file containing D1-D2–PsbE coordinates was extracted from an available PSII structure (PDB ID: 8Z9D) (15) and used as the docking receptor molecule with Rub-ENH1 (Rub domain) as a ligand. There are multiple domains in Rub-ENH1 (Fig. 6*A*), in our docking practice, we only used the redox active non-heme iron-containing domain (Rub) as the docking ligand molecule. The use of the truncated Rub-ENH1 is due to our experimental observation (Fig. 5) and prior knowledge of close association of the redox active domain of RBD1 with D1-D2 module (29, 31, 32, 48). D1-D2 stromal side is highly electrostatically negative due to the abundant glutamate and aspartate amino acid residues clustered on the solvent accessible surface (Fig. 6*B*). This is in contrast to the Rub-ENH1 which has one positively charged surface on the non-heme iron domain (Fig. 6*C*) and one negatively charged side on the other (Fig. 6*D*). In our docking practice, we considered this Coulombic forces and assigned the potential interactional interface as electrostatically favored, *i.e.,* highly negatively charged D1-D2 attracts the positively charged Rub-ENH1 domain (non-heme domain) (Fig. 6*C*). HADDOCK produced 163 structures in nine cluster(s), which represent 81% of the water-refined models HADDOCK generated (67) (Table 2). The top cluster (1) is the most reliable according to HADDOCK. The best four of the top nine clusters were then further evaluated based on the distance restraints (<30 Å) (Fig. 5, Table 4). We found that almost all of the average distances between the α-carbon of PsbE-S^2^ and Rub-ENH1_K^185^ satisfy the chemical restraint of the cross-linker with an exception of cluster three. Cluster 8 has the smallest value of distance (24.9 Å). However, it also has the highest HADDOCK score which is thermodynamically least favorable.

**Table 4:** HADDOCK docking results of Rub-ENH1 to D1/D2

Clusters	1	2	4	3	9	5	7	6	8
HADDOCK score	−98.8 ± 5.6	−94.2 ± 4.2	−80.3 ± 2.1	−70.0 ± 3.0	−69.9 ± 7.9	−63.1 ± 6.6	−62.2 ± 3.8	−54.0 ± 1.5	−43.5 ± 13.1
Cluster size	61	31	19	21	4	12	5	6	4
RMSD^a^	0.7 ± 0.5	6.8 ± 0.2	5.0 ± 0.3	8.1 ± 0.2	6.1 ± 0.2	5.7 ± 0.1	4.7 ± 0.9	3.2 ± 1.0	5.4 ± 0.2
Van der Waals energy	−34.7 ± 7.3	−22.2 ± 7.0	−22.8 ± 1.4	−7.0 ± 7.2	−15.9 ± 2.5	−31.4 ± 4.3	−11.7 ± 3.6	−11.8 ± 1.1	−9.5 ± 4.6
Electrostatic energy	−385.7 ± 20.5	−386.1 ± 21.3	−310.4 ± 19.5	−370.0 ± 35.5	−316.1 ± 30.4	−201.8 ± 41.0	−322.5 ± 15.5	−201.1 ± 8.8	−228.7 ± 46.7
Desolvation energy	10.7 ± 2.1	1.9 ± 1.7	4.0 ± 2.0	8.2 ± 2.1	5.7 ± 0.8	6.4 ± 2.4	10.9 ± 1.9	−2.9 ± 1.4	11.2 ± 2.1
Restraints violation energy	23.3 ± 13.0	33.8 ± 21.6	6.1 ± 1.8	28.1 ± 28.0	34.6 ± 12.2	22.6 ± 28.1	30.6 ± 33.3	8.7 ± 11.4	4.9 ± 4.9
Buried surface area	1303.8 ± 71.5	1064.5 ± 76.9	1035.1 ± 38.4	1025.0 ± 50.0	938.4 ± 38.5	1310.8 ± 63.6	783.2 ± 81.5	645.4 ± 74.5	937.5 ± 178.0
Z-score	−1.7	−1.4	−0.6	0	0	0.4	0.5	1	1.6
Distances (Å)^b^	26.2	28.6	29.8	32.4	29.9	27.7	30	27.7	24.9

a RMSD from the overall lowest-energy structure.

b Average distances (Å) (α-carbon).

Five representative clusters Rub-ENH1–D1-D2–PsbE complexes were show in Figure 6, *E*–*I*. All these models demonstrated that Rub-ENH1 Rub domain sits on the interface of D1-D2, possibly stabilized by multiple salt-bridging formed between negatively and positively charged side chains from D1-D2 and Rub-ENH1 Rub domain: D1-E^242/243^–Rub-ENH1-R^181^ and D2-E^241^–Rub-ENH1-R^184^. (Fig. 6*J*, cluster 1 model 1). If the whole protein Rub-ENH1 (three domains) is docked onto D1-D2, it seems that the Rub-ENH1 PDZ domain is also located on the surface of D1-D2 and the N-terminal transmembrane helix is in the membrane (not perfect in transmembrane plane of D1-D2 though). Please note that there is a loop region between the Rub domain and the single transmembrane helix domain; this may reduce the accuracy of the structural prediction for the loop, which subsequently biases the orientation of the domain on either side (Fig. 6*K*). The iron of Rub-ENH1 seemingly is located on the top of the iron ion coordinated by D1 and D2 (Fig. 6*L*).

The flexible loops/extensions on the stromal side of PSII subunits make the structural modeling erroneous. For example*,* the N-terminal extensions of PsbE (Fig. 6) on the central two monomeric PSIIs of C4S4M2 were resolved, but not the two PsbE NETs on the peripheral side of the megacomplex (15), probably due to the use of detergents and biochemical preparations that disrupt the intact structure or due to the intrinsic flexible nature of such regions. This hypothesis also applies to many loops and other terminal extensions of PSII subunits that are exposed onto the stromal side. We consider our modelings are our best effort combining the structural MS data and the computational structural protein bioinformatics. It is not our intention to overinterpret our data.

We have previously reported that PsbR’s large N-terminal domain is located on the stromal side of PSII, closely binding to D1, D2, and CP43. PsbR may particularly play important roles in regulating the plastoquinone occupancy in Q_B_ site (15). However, the occupancy of Rub-ENH1 in PSII of all of our models is spatially in conflict with that of PsbR, partially supporting our failed docking analysis if PsbR is included in the D1-D2 structure (data not shown), *i.e.,* D1-D2–PsbE–PsbR, indicating that if Rub-ENH1 is involved in PSII biogenesis, PsbR must be recruited after Rub-ENH1 leaves the binding site which may indicate that PsbR is more involved in steady-state PSII functionality; if Rub-ENH1 binds to the steady-state functional PSII as PsbR does, then, there must be different populations of PSII, *i.e.,* Rub-ENH1-containing PSII and PsbR-containing PSII. RBD1 and RubA in cyanobacteria have been demonstrated essential for PSII D1 assembly (29, 30, 31, 32, 48), *i.e.,* RBD1 is associated with PSII assembly intermediates without fully PSII oxygen evolution activity. Protein sequence alignment analysis indicated that A0A9R0ICQ3 is highly homologous to AT5G17170 (ENH1) rather than AT1G54500 (RBD1) (Figs. S1 and S2). The specific sequence between the conserved CLDC and CPQC motifs in Rub-ENH1 in *Arabidopsis* and spinach is 18-amino acid long, which is much shorter than the 29 amino acid spacer found in the “true” RBD1 (AT1G54500) in *Arabidopsis*, indicating that they do not perform the same function in the plants. Early research on Rub-ENH1 indicated the deletion mutant (At5g17170) caused enhanced accumulation of reactive oxygen species, particular under salt stress (45). Molecular evolution and functional analysis indicated that Rub proteins containing PDZ and Rub domains are localized to the chloroplast and play important roles under abiotic stress in higher plants possibly through ion homeostasis (45, 46). It is highly suggested here that future research on the roles of Rub-ENH1 in PSII’s function is needed.

## Conclusions

With applications of isotopically encoded chemical cross-linking and LC-MS/MS interrogations and powerful protein structural prediction and modeling, it was possible to more precisely locate proteins onto protein complexes. The solid data of our cross-linking pairs with isotopic features and the chemical restraints intrinsic to the cross-linkers allow us to evaluate the *in silico* models produced by modern protein docking platforms. We were able to dock Psb27-H1 onto the luminal side of PSII CP43 and further dock TLP18.3 onto CP43–Psb27 complex. All structural models have been justified by applying the distance restraint of the cross-linker. Finding of Rub family protein (Rub-ENH1) closely associated with the stromal side of PSII, particularly D1-D2 proteins, demonstrates that increased binding potential of PSII stromal side, which greatly enhances our understanding of the redox active reactions that are involved in PSII assembly or steady-state functionality and photoprotection. This platform is expected to scale up to whole thylakoid membrane and chloroplast level to holistically understand the architecture of protein machinery involved in energy harvesting and photochemical conversion and regulation.

## Experimental procedures

### Sample preparation for MS analysis

The PSII particle preparation (so-called BBY) followed the literature (74) with minor modifications (43). Chemical cross-linking (BS3-H_12_/D_12,_ Creative Molecule Inc.), reaction quenching, and MS sample processing and LC-MS/MS interrogation of the digested samples followed the method described in literature (15, 43). For PSII particle preparation quality, please refer to the literature (75) and our latest report on *Arabidopsis* (76).

### MS data analysis

The Thermo Fisher Scientific raw files (.raw) were imported into pLink software (version 3.0.16) (35). Searching parameters were as follows: enzyme was trypsin (up to three missed cleavages), 10 ppm of precursor tolerance (±20 ppm search to correct systematic errors, then ± ppm filter), 20 ppm of fragment tolerance, [600, 6000] of peptide mass, and [6, 60] of peptide length. Variable modifications were as follows: fixed modifications, carbamidomethyl [C]; variable modifications, acetyl [ProteinN-Term], oxidation of methionine (M), deamination of glutamine (N), asparagine (Q), Xlink_DSS_H_12_ (156.078644), and Xlink_DSS_D_12_ (168.153965, manually input). The false discovery rate was under 5% at peptide level (77). Isotopic pairs (MS1 and MS2) were examined manually in raw files to confirm cross-link identification (77). Manual validation of the fragment ions with isotopic features resulting from cross-linkers with light (H_12_) and heavy (D_12_) was further performed in pLink assignment.

### Structural prediction, alignment, and protein–protein docking

Spinach proteins—TLP18.3 (A0A9R0JN50), Psb27-H1(A0A9R0KAU1), and Rub (A0A9R0ICQ3)—were submitted to either swissmodel.expasy.org through uniprot.org or alphafoldserver.com without any additional restraints for TLP18.3 and Psb27-H1 and with one iron (Fe3+) for Rub in the structural prediction using AlphaFold 3.0 (68). Structural alignment was performed using PyMOL (https://www.pymol.org/). Molecular docking (protein–protein) of TLP18.3 and Psb27-H1 was performed in either HADDOCK server (67) or ClusPro server (https://cluspro.bu.edu/login.php) with or without chemistry restraints (78, 79). Ligand–receptor models were screened based on our chemistry restraints from structural MS (*e.g.,* TLP18.3-K^57^–Psb27-H1-K^92^, Table 1). The distance measurement between primary amine groups between cross-linked pairs was visualized and performed using PyMOL.

### Bioinformatic analysis of protein sequences

Homologs of the studied proteins were found by searching against nonredundant protein sequences on National Center for Biological Information BlastP separately. Sequences of the 300 to 500 proteome with a protein sequence closest to spinach were used for sequence alignments. Sequences with significant alignments were downloaded as an aligned sequence file, and the generated file was manually trimmed to remove large indels using MEGA11 (80) in line with spinach protein sequence. The trimmed file was used for visualization with Weblogo 3.7.4 (81).

## Data availability

The mass spectrometry proteomics data have been deposited to the ProteomeXchange Consortium *via* the PRIDE (82) partner repository with the dataset identifier PXD073794.

## Supporting information

This article contains supporting information.

## Conflict of interest

The authors declare that they have no conflicts of interest with the contents of this article.

## References

[1] Oliver T., Kim T.D., Trinugroho J.P., Cordon-Preciado V., Wijayatilake N., Bhatia A. (2023). The evolution and evolvability of photosystem II. Annu. Rev. Plant Biol..

[2] Nelson N., Yocum C.F. (2006). Structure and function of photosystems I and II. Annu. Rev. Plant Biol..

[3] Vinyard D.J., Brudvig G.W. (2017). Progress toward a molecular mechanism of water oxidation in photosystem II. Annu. Rev. Phys. Chem..

[4] Cao P., Pan X., Su X., Liu Z., Li M. (2020). Assembly of eukaryotic photosystem II with diverse light-harvesting antennas. Curr. Opin. Struct. Biol..

[5] Iwai M., Patel-Tupper D., Niyogi K.K. (2024). Structural diversity in eukaryotic photosynthetic light harvesting. Annu. Rev. Plant Biol..

[6] Muzzopappa F., Kirilovsky D. (2020). Changing color for photoprotection: the orange carotenoid protein. Trends Plant Sci..

[7] Ware M.A., Belgio E., Ruban A.V. (2015). Comparison of the protective effectiveness of NPQ in arabidopsis plants deficient in PsbS protein and zeaxanthin. J. Exp. Bot..

[8] Komenda J., Sobotka R., Nixon P.J. (2024). The biogenesis and maintenance of PSII: recent advances and current challenges. Plant Cell.

[9] Johnson V.M., Pakrasi H.B. (2022). Advances in the understanding of the lifecycle of photosystem II. Microorganisms.

[10] Jarvi S., Suorsa M., Aro E.M. (2015). Photosystem II repair in plant chloroplasts--regulation, assisting proteins and shared components with photosystem II biogenesis. Biochim. Biophys. Acta.

[11] Umena Y., Kawakami K., Shen J.R., Kamiya N. (2011). Crystal structure of oxygen-evolving photosystem II at a resolution of 1.9 A. Nature.

[12] Kashino Y., Lauber W.M., Carroll J.A., Wang Q., Whitmarsh J., Satoh K. (2002). Proteomic analysis of a highly active photosystem II preparation from the cyanobacterium synechocystis sp. PCC 6803 reveals the presence of novel polypeptides. Biochemistry.

[13] Zouni A., Witt H.T., Kern J., Fromme P., Krauss N., Saenger W. (2001). Crystal structure of photosystem II from synechococcus elongatus at 3.8 A resolution. Nature.

[14] Zhang X., Xiao Y., You X., Sun S., Sui S.F. (2024). In situ structural determination of cyanobacterial phycobilisome-PSII supercomplex by STAgSPA strategy. Nat. Commun..

[15] Shan J., Niedzwiedzki D.M., Tomar R.S., Liu Z., Liu H. (2024). Architecture and functional regulation of a plant PSII-LHCII megacomplex. Sci. Adv..

[16] Dominguez-Martin M.A., Sauer P.V., Kirst H., Sutter M., Bina D., Greber B.J. (2022). Structures of a phycobilisome in light-harvesting and photoprotected states. Nature.

[17] Mao Z., Li X., Li Z., Shen L., Li X., Yang Y. (2024). Structure and distinct supramolecular organization of a PSII-ACPII dimer from a cryptophyte alga chroomonas placoidea. Nat. Commun..

[18] Nickelsen J., Rengstl B. (2013). Photosystem II assembly: from cyanobacteria to plants. Annu. Rev. Plant Biol..

[19] Zabret J., Bohn S., Schuller S.K., Arnolds O., Moller M., Meier-Credo J. (2021). Structural insights into photosystem II assembly. Nat. Plants.

[20] Huang G., Xiao Y., Pi X., Zhao L., Zhu Q., Wang W. (2021). Structural insights into a dimeric Psb27-photosystem II complex from a cyanobacterium thermosynechococcus vulcanus. Proc. Natl. Acad. Sci. U.S.A..

[21] Fadeeva M., Klaiman D., Caspy I., Nelson N. (2023). Structure of chlorella ohadii photosystem II reveals protective mechanisms against environmental stress. Cells.

[22] Wang Y., Wang C., Li A., Liu Z. (2025). Roles of multiple TEF30-associated intermediate complexes in the repair and reassembly of photosystem II in chlamydomonas reinhardtii. Nat. Plants.

[23] Muranaka L.S., Rutgers M., Bujaldon S., Heublein A., Geimer S., Wollman F.A. (2016). TEF30 interacts with photosystem II monomers and is involved in the repair of photodamaged photosystem II in chlamydomonas reinhardtii. Plant Physiol..

[24] Xiao Y., Huang G., You X., Zhu Q., Wang W., Kuang T. (2021). Structural insights into cyanobacterial photosystem II intermediates associated with Psb28 and Tsl0063. Nat. Plants.

[25] Sirpio S., Allahverdiyeva Y., Suorsa M., Paakkarinen V., Vainonen J., Battchikova N. (2007). TLP18.3, a novel thylakoid lumen protein regulating photosystem II repair cycle. Biochem. J..

[26] Jarvi S., Isojarvi J., Kangasjarvi S., Salojarvi J., Mamedov F., Suorsa M. (2016). Photosystem II repair and plant immunity: lessons learned from arabidopsis mutant lacking the THYLAKOID LUMEN PROTEIN 18.3. Front. Plant Sci..

[27] Wegener K.M., Bennewitz S., Oelmuller R., Pakrasi H.B. (2011). The Psb32 protein aids in repairing photodamaged photosystem II in the cyanobacterium synechocystis 6803. Mol. Plant.

[28] Wu H.Y., Liu M.S., Lin T.P., Cheng Y.S. (2011). Structural and functional assays of AtTLP18.3 identify its novel acid phosphatase activity in thylakoid lumen. Plant Physiol..

[29] Calderon R.H., Garcia-Cerdan J.G., Malnoe A., Cook R., Russell J.J., Gaw C. (2013). A conserved rubredoxin is necessary for photosystem II accumulation in diverse oxygenic photoautotrophs. J. Biol. Chem..

[30] Calderon R.H., de Vitry C., Wollman F.A., Niyogi K.K. (2023). Rubredoxin 1 promotes the proper folding of D1 and is not required for heme b(559) assembly in chlamydomonas photosystem II. J. Biol. Chem..

[31] Che L., Meng H., Ruan J., Peng L., Zhang L. (2022). Rubredoxin 1 is required for formation of the functional photosystem II core complex in arabidopsis thaliana. Front. Plant Sci..

[32] Kiss E., Knoppova J., Aznar G.P., Pilny J., Yu J., Halada P. (2019). A photosynthesis-specific rubredoxin-like protein is required for efficient association of the D1 and D2 proteins during the initial steps of photosystem II assembly. Plant Cell.

[33] Rojas Echeverri J.C., Hause F., Iacobucci C., Ihling C.H., Tanzler D., Shulman N. (2024). A workflow for improved analysis of cross-linking mass spectrometry data integrating parallel accumulation-serial fragmentation with MeroX and skyline. Anal. Chem..

[34] Petrotchenko E.V., Borchers C.H. (2010). Crosslinking combined with mass spectrometry for structural proteomics. Mass. Spectrom. Rev..

[35] Chen Z.L., Meng J.M., Cao Y., Yin J.L., Fang R.Q., Fan S.B. (2019). A high-speed search engine pLink 2 with systematic evaluation for proteome-scale identification of cross-linked peptides. Nat. Commun..

[36] Hoopmann M.R., Shteynberg D.D., Zelter A., Riffle M., Lyon A.S., Agard D.A. (2023). Improved analysis of cross-linking mass spectrometry data with kojak 2.0, advanced by integration into the trans-proteomic pipeline. J. Proteome Res..

[37] Gotze M., Pettelkau J., Schaks S., Bosse K., Ihling C.H., Krauth F. (2012). StavroX--a software for analyzing crosslinked products in protein interaction studies. J. Am. Soc. Mass. Spectrom..

[38] Iacobucci C., Gotze M., Ihling C.H., Piotrowski C., Arlt C., Schafer M. (2018). A cross-linking/mass spectrometry workflow based on MS-cleavable cross-linkers and the MeroX software for studying protein structures and protein-protein interactions. Nat. Protoc..

[39] Mendes M.L., Fischer L., Chen Z.A., Barbon M., O'Reilly F.J., Giese S.H. (2019). An integrated workflow for crosslinking mass spectrometry. Mol. Syst. Biol..

[40] Liu H., Zhang M.M., Weisz D.A., Cheng M., Pakrasi H.B., Blankenship R.E. (2021). Structure of cyanobacterial phycobilisome core revealed by structural modeling and chemical cross-linking. Sci. Adv..

[41] Liu H., Huang R.Y., Chen J., Gross M.L., Pakrasi H.B. (2011). Psb27, a transiently associated protein, binds to the chlorophyll binding protein CP43 in photosystem II assembly intermediates. Proc. Natl. Acad. Sci. U.S.A..

[42] Albanese P., Tamara S., Saracco G., Scheltema R.A., Pagliano C. (2020). How paired PSII-LHCII supercomplexes mediate the stacking of plant thylakoid membranes unveiled by structural mass-spectrometry. Nat. Commun..

[43] Mummadisetti M., Su X., Liu H. (2023). An approach to nearest neighbor analysis of pigment-protein complexes using chemical cross-linking in combination with mass spectrometry. Methods Enzymol..

[44] Weisz D.A., Liu H., Zhang H., Thangapandian S., Tajkhorshid E., Gross M.L. (2017). Mass spectrometry-based cross-linking study shows that the Psb28 protein binds to cytochrome b(559) in photosystem II. Proc. Natl. Acad. Sci. U.S.A..

[45] Zhu J., Fu X., Koo Y.D., Zhu J.K., Jenney F.E., Adams M.W. (2007). An enhancer mutant of arabidopsis salt overly sensitive 3 mediates both ion homeostasis and the oxidative stress response. Mol. Cell Biol..

[46] Li Y., Liu P.P., Ni X. (2019). Molecular evolution and functional analysis of rubredoxin-like proteins in plants. Biomed. Res. Int..

[47] Garcia-Cerdan J.G., Furst A.L., McDonald K.L., Schunemann D., Francis M.B., Niyogi K.K. (2019). A thylakoid membrane-bound and redox-active rubredoxin (RBD1) functions in de novo assembly and repair of photosystem II. Proc. Natl. Acad. Sci. U.S.A..

[48] Rojas M., Williams-Carrier R., Chotewutmontri P., Belcher S., Boyce E., Barkan A. (2025). Opposing action of photosystem II assembly factors RBD1 and HCF136 underlies light-regulated psbA translation in plant chloroplasts. Proc. Natl. Acad. Sci. U.S.A..

[49] van Bezouwen L.S., Caffarri S., Kale R.S., Kouril R., Thunnissen A.W.H., Oostergetel G.T. (2017). Subunit and chlorophyll organization of the plant photosystem II supercomplex. Nat. Plants.

[50] Su X., Ma J., Wei X., Cao P., Zhu D., Chang W. (2017). Structure and assembly mechanism of plant C(2)S(2)M(2)-type PSII-LHCII supercomplex. Science.

[51] Caferri R., Zhou Q., Dall'Osto L., Amelii A., Shan J., Liu Z. (2025). A stress-induced paralog of Lhcb4 controls the photosystem II functional architecture in arabidopsis thaliana. Nat. Commun..

[52] Graca A.T., Hall M., Persson K., Schroder W.P. (2021). High-resolution model of arabidopsis photosystem II reveals the structural consequences of digitonin-extraction. Sci. Rep..

[53] Sinz A. (2018). Cross-Linking/Mass spectrometry for studying protein structures and protein-protein interactions: where are we now and where should we go from here?. Angew. Chem. Int. Ed. Engl..

[54] Liu H., Weisz D.A., Zhang M.M., Cheng M., Zhang B., Zhang H. (2019). Phycobilisomes harbor FNR(L) in cyanobacteria. mBio.

[55] Roose J.L., Pakrasi H.B. (2004). Evidence that D1 processing is required for manganese binding and extrinsic protein assembly into photosystem II. J. Biol. Chem..

[56] Nowaczyk M.M., Hebeler R., Schlodder E., Meyer H.E., Warscheid B., Rogner M. (2006). Psb27, a cyanobacterial lipoprotein, is involved in the repair cycle of photosystem II. Plant Cell.

[57] Cormann K.U., Moller M., Nowaczyk M.M. (2016). Critical assessment of protein cross-linking and molecular docking: an updated model for the interaction between photosystem II and Psb27. Front. Plant Sci..

[58] Komenda J., Knoppova J., Kopecna J., Sobotka R., Halada P., Yu J. (2012). The Psb27 assembly factor binds to the CP43 complex of photosystem II in the cyanobacterium synechocystis sp. PCC 6803. Plant Physiol..

[59] Liu H., Roose J.L., Cameron J.C., Pakrasi H.B. (2011). A genetically tagged Psb27 protein allows purification of two consecutive photosystem II (PSII) assembly intermediates in synechocystis 6803, a cyanobacterium. J. Biol. Chem..

[60] Liu H., Chen J., Huang R.Y., Weisz D., Gross M.L., Pakrasi H.B. (2013). Mass spectrometry-based footprinting reveals structural dynamics of loop E of the chlorophyll-binding protein CP43 during photosystem II assembly in the cyanobacterium synechocystis 6803. J. Biol. Chem..

[61] Chen H., Zhang D., Guo J., Wu H., Jin M., Lu Q. (2006). A Psb27 homologue in arabidopsis thaliana is required for efficient repair of photodamaged photosystem II. Plant Mol. Biol..

[62] Hou X., Fu A., Garcia V.J., Buchanan B.B., Luan S. (2015). PSB27: a thylakoid protein enabling arabidopsis to adapt to changing light intensity. Proc. Natl. Acad. Sci. U.S.A..

[63] Xingxing C., Jiuyang L., Huan Z., Fudong L., Shuya Z., Min X. (2018). Crystal structure of Psb27 from arabidopsis thaliana determined at a resolution of 1.85 A. Photosynth Res..

[64] Cormann K.U., Bangert J.A., Ikeuchi M., Rogner M., Stoll R., Nowaczyk M.M. (2009). Structure of Psb27 in solution: implications for transient binding to photosystem II during biogenesis and repair. Biochemistry.

[65] Mabbitt P.D., Rautureau G.J., Day C.L., Wilbanks S.M., Eaton-Rye J.J., Hinds M.G. (2009). Solution structure of Psb27 from cyanobacterial photosystem II. Biochemistry.

[66] Michoux F., Takasaka K., Boehm M., Komenda J., Nixon P.J., Murray J.W. (2012). Crystal structure of the Psb27 assembly factor at 1.6 A: implications for binding to photosystem II. Photosynth Res..

[67] Honorato R.V., Trellet M.E., Jimenez-Garcia B., Schaarschmidt J.J., Giulini M., Reys V. (2024). The HADDOCK2.4 web server for integrative modeling of biomolecular complexes. Nat. Protoc..

[68] Jumper J., Evans R., Pritzel A., Green T., Figurnov M., Ronneberger O. (2021). Highly accurate protein structure prediction with AlphaFold. Nature.

[69] Gisriel C.J., Brudvig G.W. (2022). Comparison of PsbQ and Psb27 in photosystem II provides insight into their roles. Photosynth Res..

[70] Lambertz J., Liauw P., Whitelegge J.P., Nowaczyk M.M. (2022). Mass spectrometry analysis of the photosystem II assembly factor Psb27 revealed variations in its lipid modification. Photosynth Res..

[71] Bohn S., Lo Y.K., Lambertz J., Meier-Credo J., Furtges T., L P. (2025). Structural insights into late-stage photosystem II assembly by Psb32. bioRxiv.

[72] Trinh C.S., S R., Conner W.C., Reyes A.V., Karunadasa S.S., Liu G. (2025). Mapping the architecture of protein complexes in arabidopsis using cross-linking mass spectrometry. bioRxiv.

[73] Lovenberg W., Sobel B.E. (1965). Rubredoxin: a new electron transfer protein from clostridium pasteurianum. Proc. Natl. Acad. Sci. U.S.A..

[74] Berthold D.A., Babcock G.T., Yocum C.F. (1981). A highly resolved, oxygen-evolving photosystem-II preparation from spinach thylakoid membranes - EPR and electron-transport properties. Febs Lett..

[75] Kale R., Sallans L., Frankel L.K., Bricker T.M. (2020). Natively oxidized amino acid residues in the spinach PS I-LHC I supercomplex. Photosynth Res..

[76] Niedzwiedzki D.M., Tomar R.S., Magdaong N.C.M., Liu H. (2025). Nonpigmented PsbR is involved in the integrity of excitation landscape in higher plant photosystem II, a case study in arabidopsis thaliana and a mutant. Photosynth Res..

[77] Liu H. (2023). Cyanobacterial phycobilisome allostery as revealed by quantitative mass spectrometry. Biochemistry.

[78] Kozakov D., Hall D.R., Xia B., Porter K.A., Padhorny D., Yueh C. (2017). The ClusPro web server for protein-protein docking. Nat. Protoc..

[79] Liu H. (2022). AlphaFold and structural mass spectrometry enable interrogations on the intrinsically disordered regions in cyanobacterial light-harvesting complex phycobilisome. J. Mol. Biol..

[80] Tamura K., Stecher G., Kumar S. (2021). MEGA11: molecular evolutionary genetics analysis version 11. Mol. Biol. Evol..

[81] Crooks G.E., Hon G., Chandonia J.M., Brenner S.E. (2004). WebLogo: a sequence logo generator. Genome Res..

[82] Perez-Riverol Y., Bandla C., Kundu D.J., Kamatchinathan S., Bai J., Hewapathirana S. (2024). The PRIDE database at 20 years: 2025 update. Nucleic Acids Res..

