# Supplementary material for: Structural interactions of TLP18.3 and Psb27-H1 to the luminal CP43 and rubredoxin-ENH1 to the stromal side of photosystem II in higher plants
Source: J Biol Chem. 2026 Mar 10;302(5):111363. doi: 10.1016/j.jbc.2026.111363 (PMC13089150; doi:10.1016/j.jbc.2026.111363)
Supplement: Supporting information [file mmc1.docx]

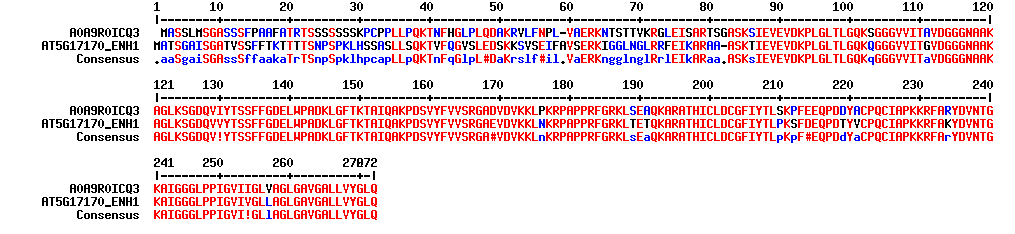


18 AA

Figure S1 Sequence alignment of A0A9R0ICQ3 (so_Rub-ENH1), Arabidopsis AT5G17170_ENH1. Conserved CLDC and CPQC are boxed in green.


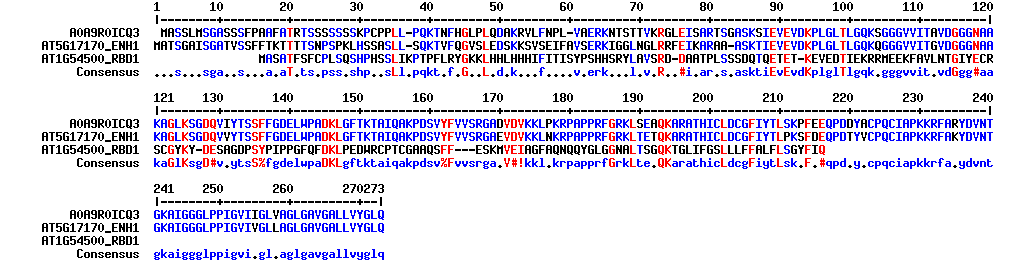


Figure S2 Sequence alignment of A0A9R0ICQ3 (so_Rub-ENH1), Arabidopsis AT5G17170_ENH1 and AT1G54500_RBD1.

Protein sequence of Rub-ENH1 and RBD1

>A0A9R0ICQ3 (spinach)

MASSLMSGASSSFPAAFATRTSSSSSSSKPCPPLLPQKTNFHGLPLQDAKRVLFNPLVAERKNTSTTVKRGLEISARTSGASKSIEVEVDKPLGLTLGQKSGGGVVITAVDGGGNAAKAGLKSGDQVIYTSSFFGDELWPADKLGFTKTAIQAKPDSVYFVVSRGADVDVKKLPKRPAPPRFGRKLSEAQKARATHICLDCGFIYTLSKPFEEQPDDYACPQCIAPKKRFARYDVNTGKAIGGGLPPIGVIIGLVAGLGAVGALLVYGLQ

>AT5G17170_ENH1(Q9FFJ2 in Uniprot.org; *Arabidopsis Thaliana*)

MATSGAISGATVSSFFTKTTTTSNPSPKLHSSASLLSQKTVFQGVSLEDSKKSVSEIFAVSERKIGGLNGLRRFEIKARAAASKTIEVEVDKPLGLTLGQKQGGGVVITGVDGGGNAAKAGLKSGDQVVYTSSFFGDELWPADKLGFTKTAIQAKPDSVYFVVSRGAEVDVKKLNKRPAPPRFGRKLTETQKARATHICLDCGFIYTLPKSFDEQPDTYVCPQCIAPKKRFAKYDVNTGKAIGGGLPPIGVIVGLLAGLGAVGALLVYGLQ

>AT1G54500_RBD1 (*Arabidopsis Thaliana*)

MASATFSFCPLSQSHPHSSLIKPTPFLRYGKKLHHLHHHIFITISYPSHHSRYLAVSRDDAATPLSSSDQTQETETKEVEDTIEKRRMEEKFAVLNTGIYECRSCGYKYDESAGDPSYPIPPGFQFDKLPEDWRCPTCGAAQSFFESKMVEIAGFAQNQQYGLGGNALTSGQKTGLIFGSLLLFFALFLSGYFIQ
